# *Lactobacillus plantarum* TWK10 Improves Muscle Mass and Functional Performance in Frail Older Adults: A Randomized, Double-Blind Clinical Trial

**DOI:** 10.3390/microorganisms9071466

**Published:** 2021-07-08

**Authors:** Mon-Chien Lee, Yu-Tsai Tu, Chia-Chia Lee, Shiow-Chwen Tsai, Han-Yin Hsu, Tsung-Yu Tsai, Te-Hua Liu, San-Land Young, Jin-Seng Lin, Chi-Chang Huang

**Affiliations:** 1 Graduate Institute of Sports Science, National Taiwan Sport University, Taoyuan City 333325, Taiwan; 1061304@ntsu.edu.tw (M.-C.L.); 1071302@ntsu.edu.tw (Y.-T.T.); 2 Department of Physical Medicine and Rehabilitation, Taipei City Hospital, Zhongxiao Branch, Taipei City 11556, Taiwan; 3 Culture Collection & Research Institute, SYNBIO TECH INC., Kaohsiung City 82151, Taiwan; cclee@synbiotech.com.tw (C.-C.L.); hanin@synbiotech.com.tw (H.-Y.H.); s333@synbiotech.com.tw (S.-L.Y.); 4 Institute of Sports Sciences, University of Taipei, Taipei City 112, Taiwan; sctsai6@gmail.com; 5 Department of Food Science, Fu Jen Catholic University, New Taipei City 24205, Taiwan; tytsai@mail.fju.edu.tw (T.-Y.T.); 147848@mail.fju.edu.tw (T.-H.L.)

**Keywords:** *Lactobacillus plantarum*, elderly, aging, muscle mass, sarcopenia, physical fragility

## Abstract

Sarcopenia is a condition in which there is a loss of muscle caused by aging and it is one of the most significant factors that affects physical fragility. In recent years, the role of the gut–muscle axis has garnered attention as, along with the gut microbiota, it potentially plays a significant role in muscle regeneration, in addition to nutritional supplements and exercise training. Past studies have found that supplementation with *Lactobacillus plantarum* TWK10 could effectively increase the muscle mass of animals or adult humans. Therefore, in this study, we investigated whether the supplementation of *L. plantarum* TWK10 produces increased muscle mass and improves the functional performance of elderly persons with mild fragility. A total of 68 elderly subjects were recruited, of which 13 subjects were excluded or withdrew from the study. We adopted a double-blind design, and the 55 subjects were randomly divided into three groups: the placebo group, the TWK10 low-dose group (2 × 10^10^ CFU/day) (TWK10-L), and the TWK10 high-dose group (6 × 10^10^ colony-forming unit (CFU)/day) (TWK10-H). For 18 weeks, all subjects were required to regularly take experimental samples, perform functional activity testing, and have their body composition analyzed before the study and every six weeks after the intervention. Finally, 17 subjects in the placebo group, 12 subjects in the TWK10-L group, and 13 subjects in the TWK10-H group finished the study. It was found that supplementation with TWK10 had a tendency to increase and improve muscle mass, left hand grip strength, lower limb muscle strength, and gait speed and balance after the sixth week, especially in the TWK10-H group, and, as the supplement time was longer up to the 18th week, it had an even greater effect (*p* < 0.05). In conclusion, consecutive supplementation of *L. plantarum* TWK10 for more than six weeks could effectively improve the muscle strength and endurance of the elderly, reducing sarcopenia and physical fragility. This trial was registered as NCT04893746.

## 1. Introduction

With the improvement of living conditions and medical technology, and with the advancement of health care concepts, global life expectancy has increased, and, worldwide, the proportion of elderly persons in the global population is gradually increasing [1]. However, an increase in average life expectancy does not necessarily mean an increase in healthy life expectancy, but also includes the persistence of various diseases and complications. Among them, the most common effect is that, during the aging process, muscle mass and strength gradually decline with age [2]. Muscle mass and strength decline at a rate of between 1% and 2% per year from the age of 50, 1.5% per year from the age of 50–60, and 3% per year thereafter, thereby significantly increasing the risk of sarcopenia [3]. The term “sarcopenia” was introduced for the first time by Rosenberg (from Greek *sarx*: flesh and *penia*: deficiency) [4]. In 2019, the actual clinical definition of sarcopenia was revised by the European Working Group on Sarcopenia in Older People 2 (EWGSOP2). The disease is a progressive and systemic skeletal muscle disease causing weaknesses that lead to falls, fractures, and physical disabilities, with an increased likelihood of consequences related to frailty and mortality [5]. Although the diagnostic evaluation criteria were different, sarcopenia may overlap significantly with physical weakness, which is one of the major factors that affects physical frailty [6]. Frailty is a multidimensional geriatric syndrome that is characterized by a cumulative decline in multiple body systems or functions [7], which affects a person’s ability to engage in daily physical activity and self-care. Additionally, it is accompanied by the gradual loss of functions or reserves of various physiological systems, resulting in negative consequences to physical, cognitive, and social abilities [8]. In view of this, physical frailty, especially the physical frailty associated with sarcopenia, is regarded as a new entity, and sarcopenia and vulnerability in the elderly were the main foci of many preventive intervention programs [9].

Many studies have shown that moderate exercise, training, or nutritional supplements could effectively prevent and reduce the risk of sarcopenia or physical frailty in the elderly [8]. Among them, exercise training mainly focuses on upper limb or lower limb muscle training that is suitable for promotion with the elderly [10,11]. For the elderly to maintain muscle mass or strength, nutritional supplements are often recommended to promote muscle synthesis and delay muscle loss, such as amino acids with a higher content of protein, vitamin D, creatine, or testosterone [12]. However, as they grow older, many elderly people not only suffer years of accumulated joint pain, inconvenience in relation to participation in exercise, or inability to effectively perform physical exercises [13], but are also prone to anorexia, as they are unable to properly eat and supplement their nutrition. As time goes by, this makes the body weaker and more susceptible to various diseases [14]. However, there is an increasing awareness of the relationship between the initial gut microbiota and human health, especially the role of the gut–muscle axis, which plays an important role in exercise performance amongst the elderly [15]. The structure of the gut microbiota changes through factors such as age, diet, antibiotic intake, disease, and other factors. Its imbalance is closely related to human health and disease and is considered to be a possible determinant of healthy aging [16]. On the other hand, it can regulate the pathophysiology of age-related sarcopenia through various mechanisms (such as inflammation and immunity, material and energy metabolism, endocrine, and insulin sensitivity), and regulate chronic inflammation and anabolic pathways to directly or indirectly affect muscle mass and function, so as to achieve the optimal “gut–muscle axis” [15,17].

Probiotic supplementation seems to be one of the most effective ways to increase and change the proportion and distribution of gut microbiota. Probiotics are defined as “living microorganisms”. When administered in appropriate amounts, probiotics can exert beneficial effects on the host by improving the function of the intestinal barrier, regulation of the immune system and cells, and the production of neurotransmitters [18]. One previous study demonstrated that, compared with subjects with lower fragility scores, subjects with higher fragility scores had significantly fewer *Lactobacillus* spp. [19]. In addition, Kaźmierczak-Siedlecka et al. found that supplementation of lactic acid bacteria (extracted from kimchi) had a dose-effective effect and significantly improved exercise performance, increased muscle mass in healthy people, and reduced muscle loss in cancer patients [20]. Therefore, *Lactobacillus* strains seem to be highly related to muscle mass and the molecular mechanisms that can improve the aging of the musculoskeletal system. Among them, *Lactobacillus plantarum* are a homofermentative, aerotolerant Gram-positive bacteria. *L. plantarum* survive in the human gastro-intestinal tract, which makes them a possible in vivo delivery vehicle for therapeutic compounds or proteins. Additionally, they demonstrate significant antioxidant activities, and also help to maintain intestinal permeability [21]. In our previous studies, both animal and human trials found that the supplementation of *L. plantarum* TWK10 for six consecutive weeks not only effectively improved exercise endurance and muscle strength performance, but also increased skeletal muscle weight [22,23]. We recently demonstrated that *L. plantarum* TWK10 supplementation attenuates aging-associated muscle weakness, bone loss, and cognitive impairment by modulating the gut microbiome in mice (Lee, CC et al. 2021). However, the current research is mainly based on animal experiments, or studies on younger adults, and the efficacy of the elderly is yet to be explored.

In the current study, we administered *L. plantarum* TWK10 long-term probiotic supplements to elderly people over 65 years old who showed mild frailty, and regularly observed the changes in their muscle strength performance, functional activity, and body composition. Through this, we explored the effect of supplementing *L. plantarum* TWK10 on improving the function promotion and body composition of the elderly.

## 2. Materials and Methods

### 2.1. Sample Preparation

TWK10 is a strain of *Lactobacillus plantarum*, which is isolated from Taiwanese pickled cabbage [22]. TWK10 was cultivated and produced in capsule form according to the specified dosage by SYNBIO TECH INC. (Kaohsiung, Taiwan). Each labeled TWK10 (lyophilized bacterial powder) capsule contains 1 × 10^10^ or 3 × 10^10^ colony-forming units (CFU) of TWK10 and was standardized with maltodextrin and microcrystalline cellulose. The composition of the placebo capsules was similar to TWK10 capsules, but TWK10 was not added.

### 2.2. Subjects

A total of 68 elderly subjects were recruited to participate in the experiment, all of whom were from Taipei City Haoran Senior Citizen Home, Department of Social Welfare, Taipei City Government, Taiwan, and they were evaluated by the rehabilitation physicians using the clinical frailty scale (CFS). Those aged 55–85 years old with frailty grades 1 to 4 were included in the study. Subjects with stroke, hypertension, and exercise contraindications confirmed by the attending physician were excluded from this study. Subjects were asked to maintain their typical routines during the experiment and to avoid taking additional probiotics, prebiotics, fermented products (yogurt or cheese), or antibiotics, in order to avoid unnecessary interference during the experiment. The study was reviewed and approved by the Institutional Review Board of Landseed International Hospital (Taoyuan, Taiwan; LSHIRB No. 19-035). All volunteers provided written, informed consent before starting the experiment.

### 2.3. Experimental Design

**Figure 1:**
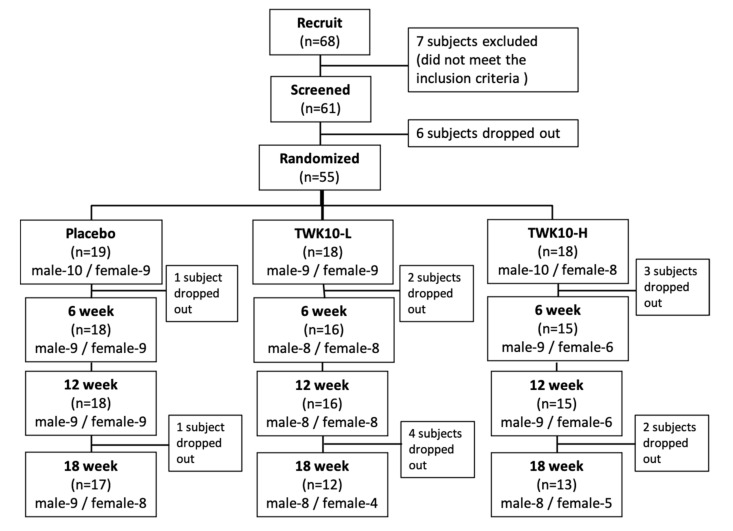
Experimental procedure description.

The test adopted a double-blind design and subjects were randomly divided into the placebo group, the TWK10 low-dose group (2 × 10^10^ CFU/day (TWK10-L), or the TWK10 high-dose group (6 × 10^10^ CFU/day) (TWK10-H). For 18 weeks, all subjects were required to regularly take the experimental samples, consuming one capsule twice daily, perform functional activity testing, and have their body composition analyzed before, and every six weeks after, the intervention. During the experiment, due to the willingness of the subjects, the inability to coordinate their participation, and other changes in their own physiological conditions, a small number of subjects in each group withdrew from the experiment. A description of the experimental procedure is provided in Figure 1.

### 2.4. Maximum Handgrip Strength Test

A Takei digital grip strength meter (T.K.K.5401, Takei Scientific Instruments Co., Ltd., Niigata, Japan) was used to measure the maximum grip strength of each hand; the unit used to measure grip strength was kilograms. Before the formal test, the subjects were required to squeeze the gripper with minimal force to ensure that it was compatible with the operating procedure or gripping distance. Researchers randomly designated the primary or non-primary hands to start the formal experiment. During the test, the subject was asked to squeeze the gripper with one hand with maximum force and repeated the hand-changing test at 60 s intervals to prevent fatigue. This exchange method was used to perform a three-repetition test, and the individual maximum grip strength of the two hands was collected as the data [24].

### 2.5. Functional Performance

This study used three tests that are suitable for elderly persons who demonstrate fragile syndrome or other similar senile diseases, and are commonly used to assess the strength, gait, and balance of the lower limb muscles [25]:

The 3 m timed up and go test: The subject is timed from the moment they get up from a chair and until they walk a distance of 3 m, walk around a pyramid, return to the chair and sit down. The test was completed twice, taking the second test for the measurement and the average value for further analysis.

The 10 m walk test: The subjects are asked to walk 16 m at the fastest speed they can manage, and the time taken to walk 10 m (closest to the center of the distance) is recorded in seconds. The 3 m at the beginning and end of the distance were not counted in this study, and a total of two averages were taken for further analysis.

The 30 s chair stand test: Each subject must sit upright on a standard chair, with their hands folded on their chest, and their feet flat on the floor. From the “start” command, the subject must quickly adopt a fully standing posture, and then sit down as quickly as possible. In this study, the action was repeated as often as possible within 30 s and the number of times subjects were able to complete the action was recorded.

### 2.6. Body Composition and Bone Mass Density (BMD)

A non-invasive dual-energy X-ray absorptive bone density testing room (Lunar iDXA, GE Healthcare, Chicago, IL, USA) was used for systemic body composition and bone density measurements. The subject was required to lie flat on the test bed, with their body at the center line, and their limbs within the detection range. Two different energy X-rays were used to scan the inspected part, then the scintillation detector received the X-rays that had penetrated the inspected part, and analyzed the obtained muscle mass, body fat, and bone density parameters through a computer.

### 2.7. Statistical Analysis

Data are expressed as the mean ± standard deviation (SD). Statistical analysis were performed using GraphPad Prism 7.04 (GraphPad Software Inc, San Diego, CA, USA). For multiple group comparisons, parametric data were analyzed by one-way ANOVA with a post hoc Tukey’s test, including grip strength, 3 m timed up and go test, 10 m walk test, muscle mass, fat mass, and bone density. A Kruskal–Wallis test was used for multiple comparisons of non-parametric data, including a 30 s chair stand test, relative muscle weight, relative fat weight, and T-score. In addition, differences within groups were analyzed by repeated ANOVA measurements (parametric data) and Friedman tests (non-parametric data) compared with baseline measurements. Differences were considered statistically significant at *p* < 0.05.

## 3. Results

### 3.1. Subject Recruitment

In total, 68 subjects were recruited. Among them, seven subjects who did not meet the inclusion criteria were excluded, and six subjects voluntarily withdrew before the start of the experiment. A total of 55 subjects were randomized and were allocated to one of the treatments. Of those, 17 subjects in the placebo group, 12 subjects in the TWK10-L group, and 13 subjects in the TWK10-H group completed the study. Table 1 shows the basic demographic profile and characteristics of subjects.

### 3.2. Effect of TWK10 Supplementation on Elderly Grip Strength

**Figure 2:**
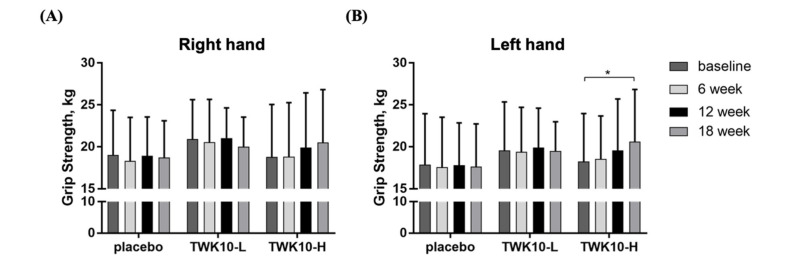
Effect of *L. plantarum* TWK10 supplementation on the (**A**) right and (**B**) left hand grip strength of elderly participants. Data are expressed as mean ± SD. * *p* < 0.05 vs. baseline.

All subjects had to take TWK10 supplements for 18 consecutive weeks, and we tested the grip strength of both of their hands every six weeks until the 18th week. As shown in Figure 2A, supplementation with TWK10 did not have a significant effect on improving the grip strength of the right hand and there were no significant differences between the placebo and TWK10 groups. The grip strengths of the left hand in the placebo, TWK10-L, and TWK10-H groups at baseline were 17.9 ± 6.1, 19.6 ± 5.8, and 18.3 ± 5.7 (kg), respectively; therefore, there were no significant differences between the groups. However, after 18 weeks of supplementation, in the placebo, TWK10-L, and TWK10-H groups, the grip strength values were 17.6 ± 5.1, 19.5 ± 3.5, and 20.6 ± 6.2 (kg). Although there were still no significant differences between each group, the left-hand grip strength of the TWK10-H group at 18 weeks was significantly (1.13-fold) higher than the baseline (*p* = 0.0187) (Figure 2B).

### 3.3. Effect of TWK10 Supplementation on Functional Performance of Elderly

**Figure 3:**
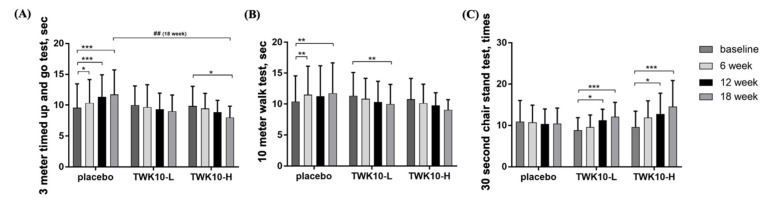
Effect of TWK10 supplementation in elderly on (**A**) 3 m timed up and go test, (**B**) 10 m walk test, and (**C**) 30 s chair stand test at baseline, 6th, 12th, and 18th weeks, respectively, in placebo group (n = 17), TWK10-L (n = 12), and TWK10-H (n = 13) groups. Data are expressed as mean ± SD. * *p* < 0.05, ** *p* < 0.01, *** *p* < 0.001 vs. baseline of each group. ## *p* < 0.01 vs. between groups at same time point.

The 3 m timed up and go test was employed to measure the elderly’s gait balance. The test times of the placebo group at the baseline, 6th, 12th, and 18th weeks were 9.4 ± 3.9, 10.3 ± 3.8, 11.4 ± 3.6, and 11.7 ± 4.0 (sec), respectively (Figure 3A). Compared with the baseline, the test time of the placebo group was significantly increased by 1.10-fold (*p* = 0.0134), 1.21-fold (*p* < 0.0001), and 1.25-fold (*p* < 0.0001) on the 6th, 12th, and 18th weeks, respectively. Supplementation with a placebo could not resist the debilitating tendency caused by aging. Supplementation with TWK10-L had no significance in terms of the outcomes of each test, but it still led to a reduced test time and a trend of improvement. The test time of the TWK10-H group at the baseline, 6th, 12th, and 18th weeks was 9.6 ± 3.2, 9.3 ± 2.5, 8.8 ± 2.0, and 8.0 ± 1.8 (sec), respectively. Although only the 18th week was significantly lower than the baseline by 16.80% (*p* = 0.0101), a steady decline overall was demonstrated. In addition, after 18 weeks of supplementation, the TWK10-H test time was also significantly lower than the placebo group by 31.66% (*p* = 0.0064). Studies have shown that supplementation with TWK10 for 18 weeks can effectively reduce the 3 m timed up and go test time and improve the gait and balance ability of the elderly.

The 10 m walk test was used to measure the walking ability and lower-limb muscle strength of the elderly. As shown in Figure 3B, supplementation with a placebo, which contained no probiotics, significantly increased the walking time in the 6th and 18th weeks compared to the baseline by 1.10-fold (*p* = 0.0013) and 1.15-fold (*p* = 0.0089), respectively. In addition, only the TWK10-L group in the 18th week had a significantly decreased walking time compared to the baseline, by 9.09% (*p* = 0.0055). On the whole, although supplementation with TWK10 had no significant improvement effect, it still had the trend benefit of maintaining and accelerating walking speed.

The 30 s chair stand test was to measure the lower-limb muscle strength and endurance of the elderly. As shown in Figure 3C, there was no significant difference in the placebo group in each test. However, the groups that received supplementation with TWK10-L or TWK10-H not only had significantly improved test times by 1.27-fold (*p* = 0.0273) and 1.33-fold (*p* = 0.0187), respectively, at the 12th week, but the effect was also more significantly increased by 1.37-fold (*p* = 0.0004) and 1.51-fold (*p* = 0.0008), respectively, at the 18th week. We believed that consecutive supplementation of TWK10 for more than 12 weeks could effectively promote and improve the muscle strength and muscular endurance performance of the lower limbs of the elderly.

### 3.4. Effect of TWK10 Supplementation on Body Composition of the Elderly

**Figure 4:**
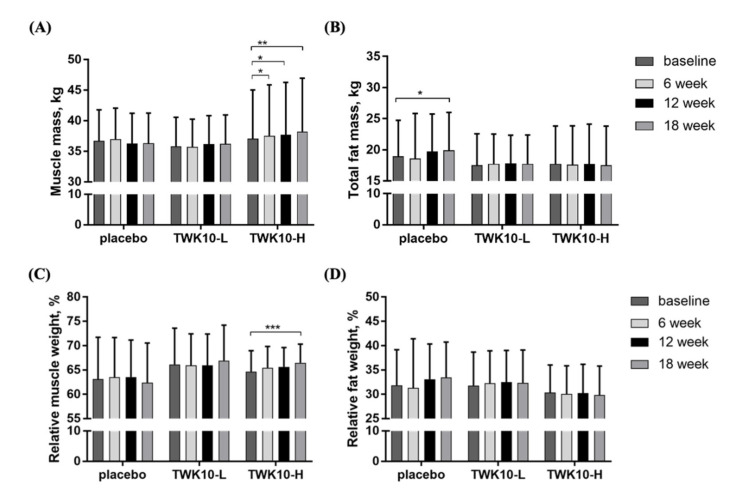
Effect of TWK10 supplementation in elderly on (**A**) muscle mass, (**B**) fat mass, (**C**) relative muscle mass, and (**D**) relative fat mass at baseline, 6th, 12th, and 18th weeks, respectively, in placebo group (n = 17), TWK10-L (n = 12), and TWK10-H (n = 13). Data are expressed as mean ± SD. * *p* < 0.05, ** *p* < 0.01, *** *p* < 0.001 vs. baseline of each group.

Muscle mass from DXA data showed (Figure 4A) no significant differences in the placebo or TWK10-L groups at each time point, compared with the baseline or between the groups. However, the muscle mass in the TWK10-H group at the baseline, 6th, 12th, and 18th weeks was 37.1 ± 8.0, 37.6 ± 8.3, 37.7 ± 8.6, and 38.2 ± 8.7 (kg), respectively. Compared with the baseline, their muscle was significantly increased at the 6th, 12th, and 18th weeks by 1.01-fold (*p* = 0.0304), 1.02-fold. (*p* = 0.0417), and 1.03-fold (*p* = 0.0020), respectively. After 18 weeks of supplementation, the placebo group showed a 1.05-fold increase in their fat mass, which was a significant increase (*p* = 0.0259) (Figure 4B).

Since tissue weight would be affected by individual weight differences, we quantified the differences between tissue weight and body weight. As shown in Figure 4C, only supplementation with TWK10-H for 18 consecutive weeks led to a significantly increased (1.03-fold (*p* < 0.0001)) relative muscle mass compared to the baseline. In addition, in terms of relative fat weight, there were no significant differences among the placebo, TWK10-L, and TWK10-H groups, or at each time point of each test compared with the baseline (Figure 4D).

### 3.5. Effect of TWK10 Supplementation on Bone Mineral Density (BMD) of Elderly

**Figure 5:**
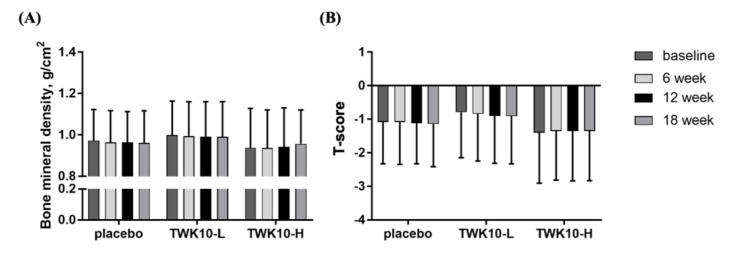
Effect of *L. plantarum* TWK10 supplementation on the (**A**) bone mineral density (BMD) and (**B**) T-score of elderly participants. Data are expressed as mean ± SD.

Through the detailed bone mineral density examination conducted by DXA, we found that, despite supplementation with TWK10-H for 18 weeks, there was a tendency toward a gradually increasing BMD. However, just like the placebo and TWK10-L groups, there were no significant differences within or between groups (Figure 5A).

The T-score is the sum of the many standard deviations between the average BMD of a patient and the average of the population compared to a reference population matched by gender and race [26]. It is used as a reference indicator for determining osteoporosis [27]. In this study, there were no significant differences within or between groups (Figure 5B).

## 4. Discussion

With the development of the global aging society, we must face the physical fragility and sarcopenia associated with aging. The relationship between the role of the gut–muscle axis and aging has gradually attracted attention. In the current study, elderly people with mild fragility were supplemented with *L. plantarum* TWK10 probiotics for 18 weeks, and their functional performance and body composition were analyzed; we found that TWK10 could effectively maintain and improve muscle mass, muscle strength, and physical activity performance.

Physical frailty and sarcopenia are multifactorial diseases that represent the involuntary loss of skeletal muscle mass and strength [5]. A longitudinal study showed that, in people around the age of 75, the loss of muscle mass was 0.64–0.7%/y for women and 0.8–0.98%/y for men. In addition, the muscle strength loss rate for men is 3–4%/y, while for women it is 2.5–3%/y; compared with females, males exhibit a greater magnitude of age-related muscle mass decline [28], mainly due to insufficient nutrition, a lack of physical activity, and endocrine system diseases, as well as age-related mechanisms that lead to the onset of sarcopenia, including inflammation, immune aging, anabolic, and oxidative stress [29,30]. The distribution and proportion of the gut microbiota seem to play an important role in the regulation of these mechanisms. The gut microbiota can be considered as a highly important metabolic or endocrine organ [31], which can not only produce metabolites with biological activity, but can also influence the synthesis and decomposition of muscle proteins by the use of several amino acids in nutrient or endogenous proteins [32]. Among them, tryptophan is the basic substrate for muscle protein synthesis and metabolism. It can stimulate the insulin-like growth factor 1/p70s6k/mTOR pathway in muscle cells and promote the expression of genes involved in myofibril synthesis [33]. Microbial by-products include endotoxins, such as lipopolysaccharide (LPS), and have the ability of LPS-related cytokines to absolutely affect protein balance (i.e., via synthesis and decomposition), while they can also induce systemic chronic inflammation and insulin resistance [34]. With age, the increase in endotoxin levels may lead to a decrease in muscle mass and, eventually, sarcopenia. However, supplementation with probiotics could increase the level of short-chain fatty acids (SCFA), such as acetic acid, propionic acid, and butyric acid. Acetate is mainly metabolized by muscle cells to produce energy [35]. In addition to its anti-inflammatory properties, butyric acid can also activate a variety of regulatory pathways, increase the production of ATP, and improve the metabolic efficiency of muscle fibers [36]. A previous study showed that butyrate administered to aging mice had the ability to inhibit histone deacetylase, thereby improving effects such as lean muscle mass and cross-sectional area [37]. In our study, the elderly who were mildly fragile were supplemented with a high-dose of *L. plantarum* TWK10, which beneficially increased their muscle mass after six weeks of high-dose supplementation (Figure 4A).

Decreased muscle strength is an important feature of physical frailty and sarcopenia, and leads to a decline in physical function related to age. For clinical purposes, a handheld dynamometer was used to measure the grip strength of the dominant side to assess muscle strength [38]. A past study showed that the grip strength performance between the dominant hand and the non-dominant hand of the elderly might differ by 5.0–5.6%. This was mainly due to the delayed loss in the ability to carry out daily activities and cope with life needs [39]. In this study, we found the grip strengths of both right and left hands showed an upward trend with supplementation of a high-dose of TWK10. It is worth noting that, after 18 weeks of supplementation with a high-dose of TWK10, the left-hand grip performance of most non-dominant hands could be significantly improved compared to before supplementation (Figure 2A,B). Insufficient muscle strength in the lower limbs can lead to a reduced gait speed and reduced physical activity, as well as an increased likelihood of physical fragility [40]. In our study, we demonstrated that no matter whether supplementation with TWK10 was given at a low or high dose for six weeks, the muscle strength of the lower limbs could be significantly improved, and, as the supplementation time increased, it had a more obvious effect on improvement (Figure 3C). Another assessment of the health status of elderly patients used was gait speed and balance as functional parameters. During aging, gait speed and balance not only depended on muscle strength and function, but also on the function of the central nervous system, which can predict the occurrence of chronic diseases, inconvenience, and death [41]. Previous studies have shown that the ability of the gut microbiota to produce neurotransmitters (including gamma-aminobutyric acid, norepinephrine, and dopamine) and regulate the production of serotonin in the host seems to be related to the composition of the microbiota and gait speed [42]. Román et al. found that the beneficial changes in the gut microbiota produced by the administration of probiotics were related to the improvement of gait speed, but not to changes in the fecal microbiota content [43]. We evaluated the functional performance of the elderly with the 3 m timed up and go and 10 m walk tests. The results showed that supplementation with TWK10 for 18 weeks could not only improve the gait stability and balance of the mildly disadvantaged elderly (Figure 3A), but also the walking speed (Figure 3B).

Sarcopenia seems to be highly correlated with osteoporosis. As muscle strength decreases, the prevalence of osteoporosis and osteoporosis increases. In a previous study of 500 women aged 60–85, compared with healthy participants, the prevalence of osteoporosis in patients with sarcopenia increased by 2.515-fold [44]. Another study found that the prevalence of osteoporosis in patients with sarcopenia increased by 7.3 times [45]. Recently, we also found that administered TWK-10 improved aged-related bone loss in aged mouse models (Lee CC et. al. 2021). However, in our study, supplementation with TWK10 for 18 weeks in elderly participants with a mild level of frailty significantly increased muscle mass, as well as improving the muscle strength and functional activity performance of the upper and lower limbs. However, this has not been found to have an effect on improved bone density (Figure 5A,B), so further studies are necessary to explore its mechanisms and its correlation.

The limitations of this study are as follows. These tests were conducted during the easing period of the COVID-19 epidemic in Taiwan, 2020. Although the epidemic situation in Taiwan was relatively moderate at that time, many tests and experimental arrangements in this study were subject to various controls. In addition, in order to ensure that the subjects had the same living conditions (e.g., same center, same diet and same exercise program), we recruited them from Taipei City Haoran Senior Citizen Home. However, each subject needed to be accompanied by one care staff. It was difficult to record the actual amounts of food intake and physical activity, as well as fecal sample collection from these elderly subjects for gut microbiota analysis, which is unknown and warrants further investigation. Moreover, as the subjects, the elderly had many uncertain factors associated with their participation in the study. They were easily affected by diseases, colds, or other conditions that caused them to withdraw from the experiment, resulting in the loss of a number of subjects toward the end.

## 5. Conclusions

In the current study, we found that elderly participants who were mildly fragile and who received *L. plantarum* TWK10 supplementation for 18 weeks showed a significantly improved hand grip strength and increased muscle mass. Moreover, in terms of the functional performance of elderly people in relation to lower limb muscle strength, gait speed, and balance, supplementation with TWK10 also had a significant improvement effect. We suggested that consecutive supplementation of *L. plantarum* TWK10 for more than six weeks could effectively improve the muscle strength and endurance of the elderly, and reduce the risk of sarcopenia and physical fragility.

## Figures and Tables

**Table 1:** Physiological characteristics of subjects.

Characters	Placebo (n = 17)	TWK10-L (n = 12)	TWK10-H (n = 13)
Age (year)	75.2 ± 7.2	77.8 ± 7.2	80.5 ± 9.4
Frailty (score)	2.9 ± 1.3	3.0 ± 1.4	2.8 ± 1.3
Height (cm)	157.0 ± 7.8	155.0 ± 6.8	158.0 ± 7.1
Weight (kg)	58.9 ± 9.1	54.6 ± 8.5	57.7 ± 13.7

All subjects were divided into three groups: placebo, TWK10-L, TWK10-H. Data are presented as mean ± standard deviation (SD).

## Data Availability

The data presented in this study are available on request from the corresponding author. The data are not publicly available due to the test sample patent and subject’s privacy and confidentiality.
